# 3D Printed Tablets (Printlets) with Braille and Moon Patterns for Visually Impaired Patients

**DOI:** 10.3390/pharmaceutics12020172

**Published:** 2020-02-19

**Authors:** Atheer Awad, Aliya Yao, Sarah J. Trenfield, Alvaro Goyanes, Simon Gaisford, Abdul W. Basit

**Affiliations:** 1 Department of Pharmaceutics, UCL School of Pharmacy, University College London, 29-39 Brunswick Square, London WC1N 1AX, UK; atheer.awad.15@ucl.ac.uk (A.A.); aliya.yao.14@ucl.ac.uk (A.Y.); sarah.trenfield.16@ucl.ac.uk (S.J.T.); s.gaisford@ucl.ac.uk (S.G.); 2 FabRx Ltd., 3 Romney Road, Ashford, Kent TN24 0RW, UK; 3 Departamento de Farmacología, Farmacia y Tecnología Farmacéutica, I + D Farma Group (GI-1645), Universidade de Santiago de Compostela, 15782 Santiago de Compostela, Spain

**Keywords:** three-dimensional printing, 3D printed drug products, orally disintegrating tablets, personalised medicines, personalized pharmaceuticals, blindness, visual deprivation, touch-reading compliance, tactile patterns, sight loss

## Abstract

Visual impairment and blindness affects 285 million people worldwide, resulting in a high public health burden. This study reports, for the first time, the use of three-dimensional (3D) printing to create orally disintegrating printlets (ODPs) suited for patients with visual impairment. Printlets were designed with Braille and Moon patterns on their surface, enabling patients to identify medications when taken out of their original packaging. Printlets with different shapes were fabricated to offer additional information, such as the medication indication or its dosing regimen. Despite the presence of the patterns, the printlets retained their original mechanical properties and dissolution characteristics, wherein all the printlets disintegrated within ~5 s, avoiding the need for water and facilitating self-administration of medications. Moreover, the readability of the printlets was verified by a blind person. Overall, this novel and practical approach should reduce medication errors and improve medication adherence in patients with visual impairment.

## 1. Introduction

Visual impairment and blindness are global health issues that pose substantial financial burdens for governments across the globe. In 2013, the total economic cost of vision loss in the United Kingdom (UK) was estimated at £15.8 billion [1]. Recent data state that the global prevalence of visual impairment was estimated at 285 million, of which 39 million are blind [2]. In particular, approximately 82% of all blind people are aged 50 years and over and are usually dependent on multiple medications [3]. As such, managing multiple medications on a daily basis can be difficult, especially if it involves the treatment of chronic conditions where elderly patients are even more likely to require help from a carer [4]. In turn, this could pose a serious problem in terms of patient compliance, potentially resulting in poor medication adherence, which could lead to poor treatment management, reduced therapeutic efficacy and subsequent hospitalisations. Previous studies revealed that one of the leading causes of medication non-adherence was impaired vision, wherein approximately 97% of patients with visual impairment have difficulties reading medication labels, even in the presence of optical aids, and around 24% have difficulties in distinguishing medications [4,5]. Moreover, patients with visual impairment are twice more likely to need assistance with medicine management, wherein one-third of the patients will require continual support for medication administration.

Currently, Braille is the universal tactile writing system used by the blind. It was invented by Louis Braille in 1825 and consists of up to six raised dots (height = 0.5 mm, space in between dots = 2.5 mm) in specific arrangements that represent different letters of the alphabet [6,7]. Another commonly used system is the Moon system, which was developed by William Moon in 1845. It is based on Latin Roman letters produced using raised shapes that are similar to the standard alphabets [8]. Compared to Braille, the Moon system is often considered easier to learn, especially for those who are not familiar with the raised dot patterns [9]. Currently, one approach to help visually impaired patients with medicine identification is the inclusion of Braille on the packaging of a medicinal product. Nonetheless, due to the absence of standardised packaging, patients often struggle to identify medications, especially in the case of generic medicines that could have an entirely different appearance and/or tactile sense (e.g., different size, shape, and/or colour of tablet and different package). Moreover, identification through pharmaceutical packaging becomes less useful when the patient removes the medicine out of its original container [4].

To overcome the issues with inaccurate identification of medication labels or doses by visually impaired patients, it is essential to develop a direct, standardised and cost-efficient method of incorporating Braille or Moon patterns on drug products themselves. In this regard, three-dimensional (3D) printing may offer an elegant and practical solution to the problem. 3D printing is an additive manufacturing technique in which an object is built up in a layer-by-layer manner [10,11,12,13,14], based on a 3D model designed using computer-aided design (CAD) software [15,16,17,18,19]. At present, 3D printing is often used to produce engineering prototypes due to its fast production speed and cost-effectiveness [20,21,22,23]. In the field of drug delivery, diverse constructs were already prepared using 3D printing ranging from drug-eluting implants [24,25,26,27] to medical devices [28,29,30], and personalised solid oral dosage forms [31,32,33,34,35,36,37,38,39]. In particular, 3D printing was shown to offer novel solutions to problems faced by specific patient groups [40,41,42,43,44]. For instance, a study evaluating the effect of shape and size on picking and swallowing of 3D printed tablets (also known as printlets) showed that certain new designs may be better accepted than conventional shapes [45]. More recently, a study concerning paediatric patients with a rare metabolic disease (maple syrup urine disease, MSUD) [46] indicated that 3D printing provides a viable approach to fabricate tailored medicines on-demand [47], improving acceptability and efficacy of treatment.

Selective laser sintering (SLS) is a powder-based 3D printing technology in which a laser beam is used to selectively bind powder particles together to create 3D objects [48,49,50]. SLS 3D printing is used in the fields of tissue engineering to produce scaffolds [51,52,53,54,55], and drug delivery to fabricate printlets with different release characteristics such as orally disintegrating [56,57,58], immediate release and modified release dosage forms [59]. Due to the high resolution of the laser, novel structures, such as 3D-gyroid lattices, bi-layer printlets [60] and dual miniprintlets [61], can be easily fabricated using different polymers, which could be engineered to exhibit customised drug release profiles tailored to individual patient needs. Thus, the aim of this study was to introduce a novel and practical approach for making dosage forms suited for patients with visual impairment. As such, SLS 3D printing was used to fabricate orally disintegrating printlets (ODPs) with Braille and Moon patterns. In doing so, patients could utilise these tactile patterns to identify medications, especially when they were taken out of their original packaging. Moreover, as these printlets are designed to disintegrate rapidly in the mouth, they do not require the co-administration of water. As such, this encourages self-administration of medicines, improving patient compliance and treatment efficacy.

## 2. Materials and Methods 

### 2.1. Materials

Paracetamol, United States Pharmacopeia (USP) grade (Sigma-Aldrich, Poole, UK), was used as a model drug. Kollidon VA64 (BASF, Ludwigshafen, Germany), a vinylpyrrolidone-vinyl acetate copolymer having a molecular weight of around 45,000 Da with instant release properties, was used for SLS printing. Candurin^®^ Gold Sheen, which is a pharmaceutical pigment, was purchased from Merck, Darmstadt, Germany. The Braille and Moon alphabet cards were provided as a reference by the Royal National Institute of the Blind (RNIB) in the UK. 

### 2.2. Preparation of Powder Formulation for SLS 3D Printing

A 100 g blend of 92% Kollidon VA64, 5% paracetamol and 3% Candurin^®^ Gold Sheen was prepared using a mortar and pestle until homogeneously mixed. This powder mixture was selected for printing due to its good printability and fast disintegration properties [56]. Candurin^®^ Gold Sheen was added into the formulation as an absorbent to yield an optimum sintering process. This is because the powder mixture absorbs the maximal amount of energy from the laser beam at this wavelength (445 nm). 

### 2.3. SLS 3D Printing 

**Figure 1:**
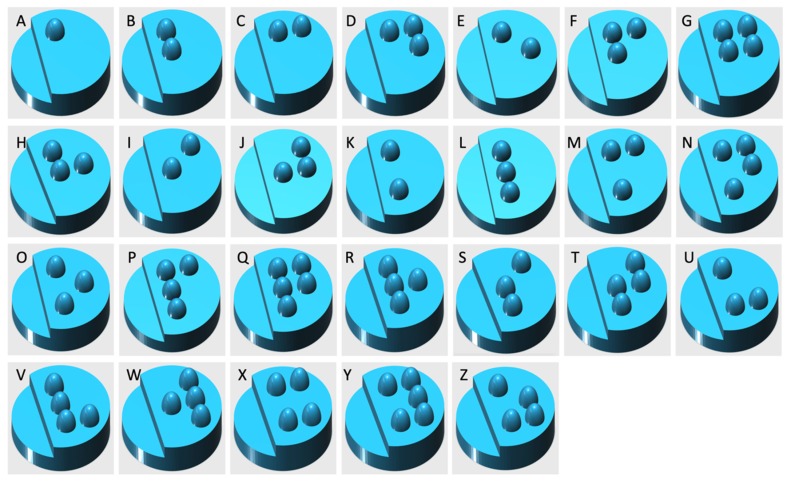
The three-dimensional (3D) models of cylindrical printlets containing the Braille alphabets.

**Figure 2:**
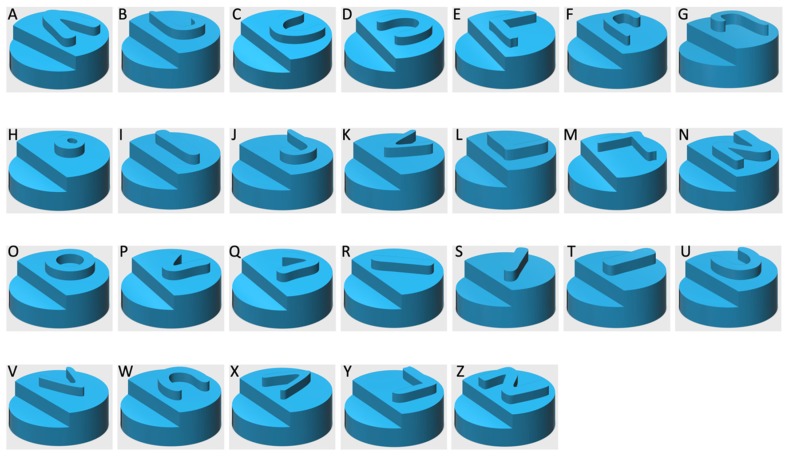
The 3D models of cylindrical printlets containing the Moon alphabets.

The homogeneous powder blend was transferred to a desktop SLS printer (Sintratec Kit, AG, Brugg, Switzerland). The 123D Design software (Version 14.2.2, Autodesk Inc., San Rafael, CA, USA) was used to create the templates for the cylindrical printlets (10 mm diameter × 3.6 mm height) with hemisphere Braille patterns (1.5 mm diameter × 0.6 mm height) or Moon alphabets (1 mm height, length ranged between 1.5–5 mm and width ranged between 2–7 mm), caplet-shaped printlets (17 mm length × 8 mm width × 6 mm height) with hemisphere Braille letter P, heart-shaped printlets (10 mm length × 10 mm width × 3.6 mm height) with Moon alphabet C (4 mm length × 3 mm width × 1 mm height), moon-shaped printlets (12 mm length × 16 mm width × 3.6 mm height) with Moon alphabet N (5 mm length × 5 mm width × 1.5 mm height), sun-shaped printlets (15 mm length × 14 mm width × 3.6 mm height) with Moon alphabet M (5.5 mm length × 4 mm width × 1 mm height), pentagon-shaped printlets (10 mm length × 10 mm width × 3.6 mm height) with Braille letter M, and square-shaped printlets (10 mm length × 10 mm width × 3.6 mm height) with Braille letter N. A caplet-shaped printlet having three Braille letters, namely, P, A, and R, was also designed (21 mm length × 8.5 mm width × 6 mm height). In the case of Braille patterns, the distance between dots in the same letter was 2.5 mm (measured centre to centre). In the case where more than one Braille letter was printed onto the same printlet, the distance between corresponding dots in adjacent letters was 6 mm. These dimensions were in accordance with the standards imposed by the UK Association for Accessible Formats (UKAAF) [62]. Images of the 3D designs of the Braille and Moon printlets are shown in Figure 1 and Figure 2. All the 3D models were exported as stereolithography (.stl) files and imported into the 3D printer Sintratec Central software (Version 1.1.13, Sintratec, AG, Brugg, Switzerland).

The powder was placed into the reservoir platform (150 × 150 × 150 mm) of the printer and was subsequently spread over to the building platform (150 × 150 × 150 mm) by a sled, yielding a flattened layer of powder. The printer was then heated, where 100 °C and 80 °C were selected as the surface and chamber temperatures, respectively. Once the set temperatures were attained, a 2.3-W blue diode laser (wavelength 445 nm) was actuated, sintering the powder particles in the building platform based on the .stl file that was imported. The laser speed was set at 300 mm/s. As each layer was sintered (layer height 100 microns), the reservoir platform moved up while the building platform moved down, and the sled spread the next layer of powder on top of the previous one. This process was repeated layer-by-layer until the printlets were fully printed. Upon completion of the printing process, the printlets were removed from the powder bed and excess powder was carefully brushed off using a fine brush. Ten printlets were printed in the same print job. A blind member of staff at the Royal National Institute of the Blind (RNIB) verified the readability of the printlets. 

### 2.4. Characterisation of the Printlets 

#### 2.4.1. Determination of the Printlet Morphology

The average weights of six printlets from each letter were measured, where the percentage of weight differences due to the addition of each letter was calculated.

#### 2.4.2. Determination of Printlet Breaking Force

The breaking forces of six printlets from each type, including printlets without patterns, printlets with Braille letter A and printlets with Braille letter Q, were determined using a traditional tablet hardness tester (TBH 200, Erweka GmbH, Heusenstamm, Germany). The process entailed the application of an increasing force perpendicular to the printlet axis on its opposite sides until it fractured.

#### 2.4.3. Scanning Electron Microscopy (SEM)

Surface images of the Braille and Moon patterns of the letter O were obtained using a scanning electron microscope (SEM, JSM-840A Scanning Microscope, JEOL GmbH, Freising, Germany). All the samples were coated with carbon (~30–40 nm).

#### 2.4.4. In Vitro Dissolution Testing 

Drug release profiles of the printlets in the presence and absence of the patterns were obtained using a USP II dissolution apparatus (PTWS 100, Pharmatest, Hainburg, Germany). For each test, the printlets (*n* = 3) were placed in 900 mL of 0.1 M HCl (pH 1.2), simulating gastric conditions. The test conditions were set at a paddle speed of 50 rpm and a temperature of 37 ± 0.5 °C. The percentage of drug released was calculated from the absorbance measured at 244 nm using an in-line ultraviolet (UV) spectrophotometer (Cecil 2020, Cecil Instruments Ltd., Cambridge, UK). 

For comparison between dissolution profiles of the printlets with and without added patterns, an ƒ_2_ similarity factor was calculated to determine how similar the dissolution profiles were for printlets with and without added patterns. The equation for calculating the ƒ_2_ similarity factor was proposed by Moore and Flanner, as shown below (Equation (1)).

(1) $$f_{2}=50\log\left\{{\left[1\;+\;\frac{1}{n}\overset{n}{\underset{n-1}{\sum }}{\left(R_{t}\;-\;T_{t}\right)}^{2}\right]}^{-0.5}\;\times \;100\right\},$$

where ƒ_2_ is a similarity factor, *n* refers to the number of observations, *Rt* is the average percentage of drug released from the reference printlets, and *Tt* is the average percentage of drug released from test printlets [63].

It was proposed that, for two dissolution profiles to be considered similar, a similarity factor of or greater than 50 (e.g., 50–100) should be attained [64]. 

#### 2.4.5. Disintegration Testing 

The disintegration times of the printlets were determined. The test was performed using a glass petri dish (100 mm × 15 mm) containing 20 mL of distilled water maintained at 37 ± 0.5 °C. A printlet was placed into the petri dish and the time needed for it to completely disintegrate was recorded. Six printlets from each type, including printlets without patterns, printlets with Braille letter A, and printlets with Braille letter Q, were evaluated.

### 2.5. Statistical Analysis

One-way ANOVA was used to evaluate whether the differences in breaking force and disintegration time of the printlets with or without the Braille patterns were statistically significant or not (*p* < 0.05) (OriginPro 2019, OriginLab corporation, Northampton, MA, USA).

## 3. Results

**Figure 3:**
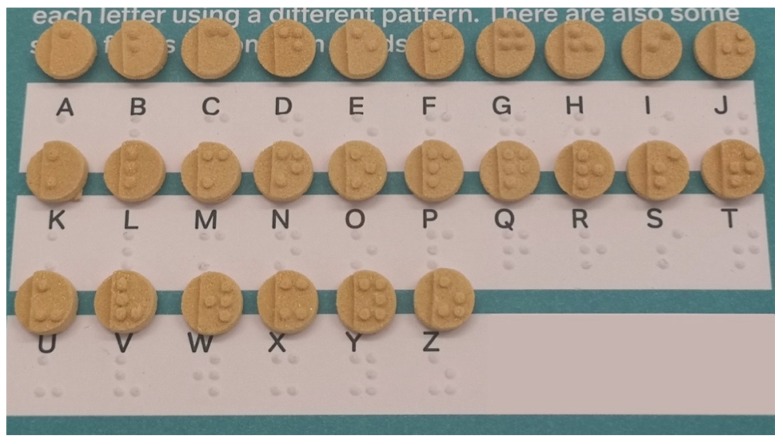
Image of cylindrical printlets containing the 26 Braille alphabets.

**Figure 4:**
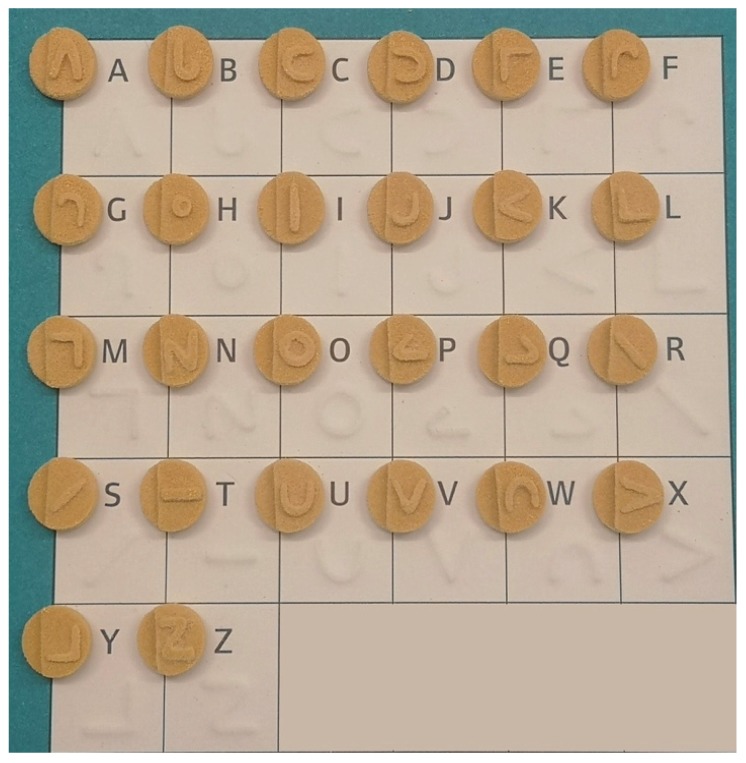
Image of cylindrical printlets containing the 26 Moon alphabets.

In this work, the SLS 3D printing technique was successfully used to print Braille and Moon patterns on the surface of cylindrical printlets, with the aim of creating personalised solid oral dosage forms that are specifically targeted to patients who are blind or visually impaired. All 26 alphabets in the form of both Braille and Moon alphabets were printed as presented in Figure 3 and Figure 4. The average weight of the Braille printlets was 171.3 mg, where the weights ranged from 164.1 ± 1.6 mg (average weight of printlets with one Braille dot) to 178.1 ± 5.6 mg (average weight of printlets with five Braille dots). In general, the addition of one Braille dot resulted in a 3.8% increase in the average weight of the printlet. For the Moon printlets, the average weight was 165.8 mg, where the weights ranged from 162 ± 1.7 mg (average weight of printlets with the letter H) to 171.1 ± 5.9 mg (average weight of printlets with the letter N). The addition of the Moon patterns resulted in an average increase of 4.9% in the average weight of the printlets, where the percentages ranged from 2.5% (for the letter H) to 8.2% (for the letter N). The patterns were visible by eye and capable of tactile recognition. The recognition was verified by a blind member of staff at the RNIB. Hence, this demonstrated another unique application of SLS in fabricating very small and detailed 3D structures that are otherwise not feasible to produce using conventional manufacturing procedures. 

The above images successfully demonstrated the ability of SLS 3D printing to produce printlets with intricate and complex patterns, which could potentially offer a new solution to improving medication adherence and independence amongst visually impaired patients. Most of the recent strategies that have been developed to help these patients are based on audio labellers such as voice-scanning devices and audible monitors, but the cost of these devices is high [65]. In 2002, it was estimated that audio descriptive devices cost the National Health Service (NHS) in the UK almost £2 million/year [66]. Hence, the novel 3D-printed dosage forms with Braille and/or Moon patterns could provide a useful and cost-efficient solution since these printlets can be manufactured in a single step to aid patient recognition of a drug. As such, the need for additional machinery or processes is avoided.

**Figure 5:**
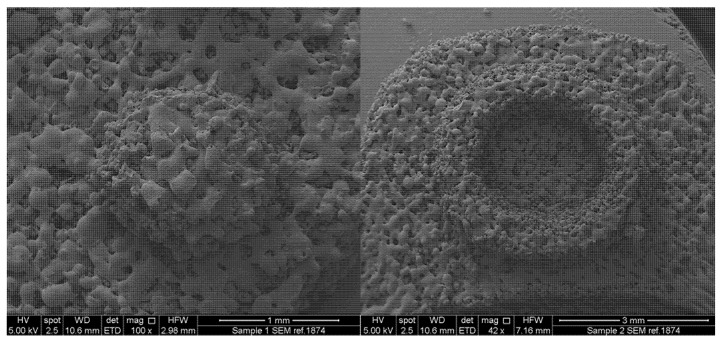
Scanning Electron Microscopy (SEM) images of (**left**) a Braille dot and (**right**) Moon alphabet of the letter O printlets.

Kollidon VA64 was selected as the main polymer matrix due to its good printability and fast disintegration properties as shown in our previous work [56]. The printlets were designed and fabricated to incorporate a small step-down from their left side. As advised by the RNIB, the purpose of this feature was to inform patients the correct direction of reading of the Braille and Moon alphabets (e.g., from left to right), thereby preventing confusion between different letters that have similar patterns. SEM imaging was performed to visualise the microstructure of the polymer particles following their sintering (Figure 5). Results show that, in both the Braille and Moon patterns, Kollicoat IR underwent a low-intensity sintering process. This can be inferred from the distinct particles that can be seen on the surfaces [60]. 

**Figure 6:**
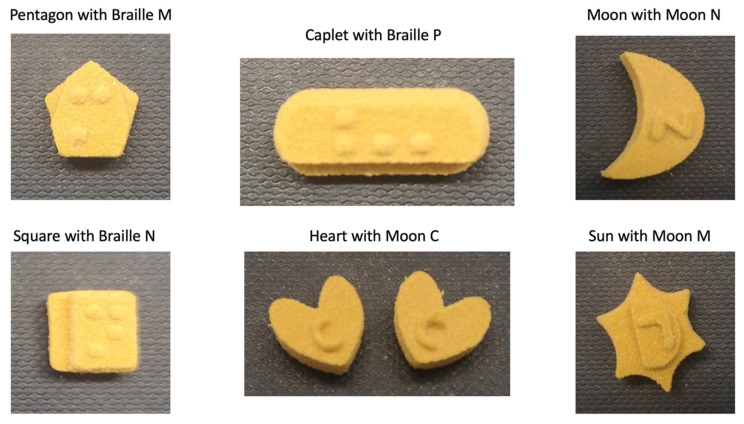
Printlets with different shapes having Braille or Moon patterns.

Printlets with novel shapes, such as a sun, a moon, a heart, a caplet, a pentagon and a square, were also successfully fabricated using SLS 3D printing (Figure 6). The aim of these shapes was to offer additional medication information to patients with low vision (e.g., medication indication and/or dosing regimen). Herein, different shapes were coupled with Braille or Moon patterns to provide additional information. The Braille and Moon letters for “M” (e.g., morning), “N” (e.g., night), “C” (e.g., cardiovascular), and “P” (e.g., paracetamol) were selected as model alphabets. A caplet shape was chosen to represent paracetamol because many commercial paracetamol products today are sold as caplets. The heart shape was selected for “cardiovascular” products simply as a direct representation of the organ that is affected. The sun and moon shapes were chosen as representations of the “morning” and “evening” dosing, respectively. Lastly, the pentagon and square shapes were chosen to differentiate the time of medicine intake because of a difference in the number of edges (e.g., number of edges can correspond to the time of intake). Unlike the other shapes, the moon and heart shapes did not require the addition of a step-down. This is mainly due to their unique configurations, wherein the right and left sides could be inferred by locating the curved top and pointed bottom of the heart. In the case of the moon shape, its inner and outer curves could be easily identified as left and right directions when the appropriate instructions are given to the patient.

**Figure 7:**
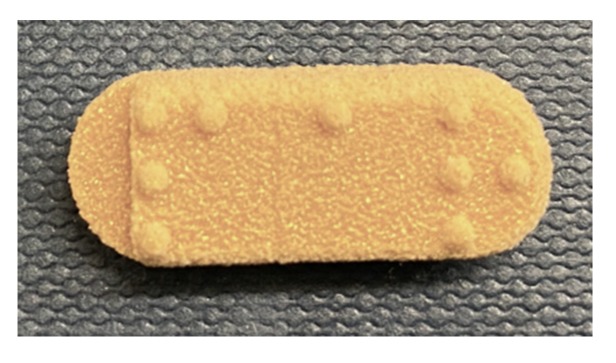
Printlet with three Braille letters, including (from left to right): P, A, and R.

A caplet containing three Braille letters was also designed, further expanding the possibilities with this technology and showing that three-letter abbreviations could be printed onto bigger-sized formulations (Figure 7). Herein, PAR was used as an abbreviation for paracetamol. The distances between the adjacent letters were designed as per the requirements of the UKAAF, wherein the standard distance between two dots within the same letter should be 2.5 mm and the standard distance between the corresponding dots in adjacent letters should be 6 mm [62]. To allow enough space for the inclusion of all the letters, one side of the caplet was designed to be flat. 

The mechanical properties of the printlets with or without the Braille patterns were assessed (Table 1). The addition of the patterns did not affect the mechanical properties of the printlets and all the printlets had similar breaking force values. A further confirmation was obtained from the statistical analysis, where no differences were seen between the groups. Consequently, the disintegration time of the printlets was assessed. In our previous work, we identified that the use of a conventional disintegration apparatus is not suitable for testing these printlets; as such, the petri dish test was used instead [56]. This is mainly because the formulations disintegrated too rapidly and, thus, conventional tests were not useful to evaluate their disintegration properties. The petri dish test was based on the Spritam^®^ disintegration test [67]. Results show that all the printlets had similar disintegration times, where no differences were seen between the groups in the statistical analysis. As these printlets disintegrate within 5 s, they are expected to dissolve in the mouth before swallowing the formulation. As shown in our previous work, the rapid disintegration is due to the high laser scanning speed which results in loose powder particle connections and porous structures [56]. 

**Figure 8:**
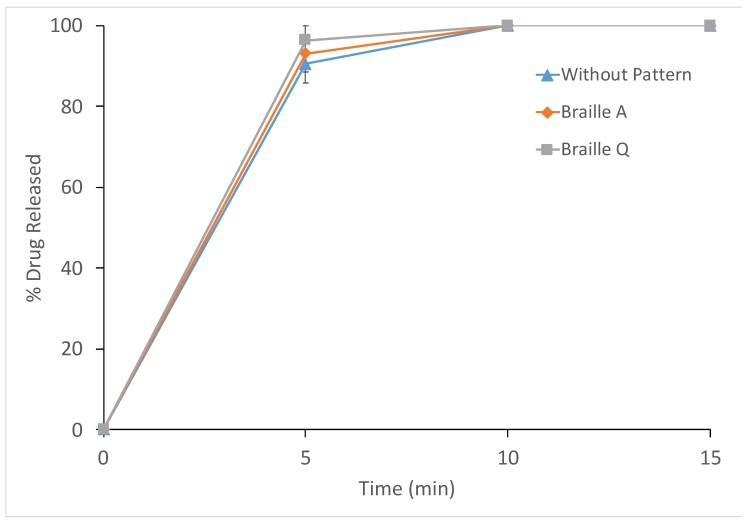
Drug dissolution profiles of the printlets (▲) without pattern, (◆) with Braille A, or (■) with Braille Q in a 0.1 M HCl (pH = 1.2) dissolution medium.

Dissolution studies were also performed using printlets having no pattern and printlets with Braille letters A and Q. The printlets were dissolved in simulated gastric fluids, and the results are shown in Figure 8. The addition of the Braille patterns did not have significant effects on the release rates of the printlets, and the drug release profile in the presence of the Braille patterns was considered similar to the release profile of the reference printlets (no pattern), where ƒ_2_ similarity values of 84 and 69 were obtained in the presence of the Braille A and Braille Q patterns, respectively. 

Many challenges are faced by visually impaired patients, including taking incorrect doses, difficulty in remembering instructions, missing a dose, taking the wrong medicine, and difficulty in identifying separate drug containers [68,69,70]. As such, most patients with visual impairment have difficulties reading medication labels, even in the presence of low-vision aids and equipment [4]. The challenges are more pronounced in the case of generic medicines, wherein the tablets and their packaging could significantly differ, leading to patient confusion. In addition to issues of low medication adherence, other costs of visual impairment extend to include intangible effects such as loss of independence, depression, emotional stress, limitation in daily life activities, and excess morbidity [33], all of which can lead to a poor quality of life, especially for the elderly. Increasing dependence on others and the increase in anxiety relating to medication management are the two most common factors leading to medication non-adherence in visually impaired elderly populations. 

The use of 3D printing provides a quick and cost-efficient method of incorporating Braille and/or Moon patterns onto drug formulations [41,71], without the need for additional machinery or steps to allow visually impaired patients to identify medications independently. Favourably, this technology offers the added benefit of using different shapes that could be inferred to a medication’s name, timing of intake (e.g., morning/evening), or its targeted indication (e.g., cardiovascular drugs). More importantly, as the pattern is directly printed on top of the tablet, the medication could be easily identified even when taken out of the packaging. This decreases the risk of medication errors and improves adherence to treatment. In addition, as these printlets disintegrate rapidly (e.g., within ~5 s), they avoid the need for water. This makes it easier for these patients to swallow the formulations, supporting self-administration and thus avoiding the need of a carer. 

## 4. Conclusions

For the first time, this study demonstrates the use of 3D printing to fabricate personalised dosage forms targeted to blind or visually impaired individuals. The SLS 3D printing technique could be used to manufacture printlets with Braille or Moon patterns on their surface that could be read by blind individuals. It is likely that this innovative concept will provide a revolutionary approach for the treatment of visually impaired individuals, improving independence, medicine adherence and reducing medicine errors. 

## Figures and Tables

**Table 1:** Mechanical properties and disintegration times of the printlets with or without the addition of the Braille patterns.

Printlet Type	Breaking Force (N ± SD)	Disintegration Time (s ± SD)
Printlets without pattern	14.5 ± 1.8	4.0 ± 1.3
Printlets with Braille A	13.9 ± 1.4	4.3 ± 1.5
Printlets with Braille Q	14.3 ± 2.1	5.2 ± 1.2
